# Impact of Enrichment and Repeated Mixing on Resilience in Pigs

**DOI:** 10.3389/fvets.2022.829060

**Published:** 2022-03-24

**Authors:** Lu Luo, Lisette E. van der Zande, Manon A. van Marwijk, Egbert Frank Knol, T. Bas Rodenburg, J. Elizabeth Bolhuis, Severine P. Parois

**Affiliations:** ^1^Adaptation Physiology Group, Department of Animal Sciences, Wageningen University & Research, Wageningen, Netherlands; ^2^Topigs Norsvin Research Center B.V., Beuningen, Netherlands; ^3^Animals in Science and Society, Faculty of Veterinary Medicine, Utrecht University, Utrecht, Netherlands; ^4^PEGASE, INRAE, Institut Agro, Saint-Gilles, France; ^5^Epidemiology Health and Welfare Research Unit, Ploufragan-Plouzané-Niort Laboratory, French Agency for Food, Environmental and Occupational Health and Safety (ANSES), Ploufragan, France

**Keywords:** resilience, enrichment, regrouping, mixing, challenge, allostatic load, pigs, chronic stress

## Abstract

Resilience, the capacity of animals to be minimally affected by a disturbance or to rapidly bounce back to the state before the challenge, may be improved by enrichment, but negatively impacted by a high allostatic load from stressful management procedures in pigs. We investigated the combined effects of diverging environmental conditions from weaning and repeated mixing to create high allostatic load on resilience of pigs. Pigs were either exposed to barren housing conditions (B) from weaning onwards or provided with sawdust, extra toys, regular access to a “play arena” and daily positive human contact (E). Half of the pigs were exposed to repeated mixing (RM) and the other half to one mixing only at weaning (minimal mixing, MM). To assess their resilience, the response to and recovery from a lipopolysaccharide (LPS) sickness challenge and a Frustration challenge were studied. In addition, potential long-term resilience indicators, i.e. natural antibodies, hair cortisol and growth were measured. Some indications of more favorable responses to the challenges in E pigs were found, such as lower serum reactive oxygen metabolite (dROM) concentrations and a smaller area under the curve of dROM after LPS injection. In the Frustration challenge, E pigs showed less standing alert, escape behaviors and other negative behaviors, a tendency for a smaller area under the curve of salivary cortisol and a lower plasma cortisol level at 1 h after the challenge. Aggression did not decrease over mixings in RM pigs and was higher in B pigs than in E pigs. Repeated mixing did not seem to reduce resilience. Contrary to expectations, RM pigs showed a higher relative growth than MM pigs during the experiment, especially in the week of the challenges. Barren RM pigs showed a lower plasma cortisol concentration than barren MM pigs after the LPS challenge, which may suggest that those RM pigs responded less detrimentally than MM pigs. Enriched RM pigs showed a higher level of IgM antibodies binding keyhole limpet hemocyanin (KLH) than enriched MM and barren RM pigs, and RM pigs showed a sharper decline in IgG antibodies binding Bovine Serum Albumin (PC-BSA) over time than MM pigs. Hair cortisol concentrations were not affected by enrichment or mixing. To conclude, enrichment did not enhance the speed of recovery from challenges in pigs, although there were indications of reduced stress. Repeated as opposed to single mixing did not seem to aggravate the negative effects of barren housing on resilience and for some parameters even seemed to reduce the negative effects of barren housing.

## Introduction

Pigs are frequently exposed to various stressful conditions in current commercial husbandry, such as abrupt weaning, transport, and mixing/regrouping. Their ability to deal with such challenges and their speed to recover, i.e. resilience, impacts their health and welfare (1). Resilience can be defined as the capacity of animals to be minimally affected by a disturbance or to rapidly bounce back to the state before the challenge, reflected as a lower sensitivity or better adaptability to the challenge (1). The slow recovery rate of animals with poor resilience might put them at risk to develop behavioral and health problems (2, 3).

Recent studies indicate that, apart from potential genetic influences (4–6), the environmental conditions under which pigs are reared and kept may influence their resilience. There are indications that resilience can be improved by better meeting the essential needs of animals, for instance, by providing enrichment (1, 7, 8). Enrichment can be defined as “an increase of the biological relevance of captive environments by appropriate modifications resulting in an improvement of the biological functioning of captive animals” (9). In support of a potential beneficial effect of enrichment on resilience, van Dixhoorn et al. (10) found that enrichment from birth onwards in the form of rooting materials, early access to non-littermates and extra space enhanced the recovery from an infectious lung challenge in pigs. Furthermore, it has been shown that piglets raised in a multi-suckling system with gradual weaning and provided with rooting materials and extra space were more resilient to several challenges that occur in pig husbandry practice, such as transport and sickness^1^.

However, most pigs on commercial farms are exposed to rather stimulus-poor, barren housing conditions that poorly meet their behavioral needs, which may negatively influence their resilience. On top of these suboptimal housing conditions, which appear to be stressful for pigs (11), the effects of other stressors pigs are exposed to, may also contribute to this. Accumulation of such stressful management procedures can cause allostatic load (12), i.e. the burden of cumulative wear and tear on the body due to adapting to adverse situations (13, 14). This allostatic load may undermine the resilience of animals to future challenges (3). It can therefore be hypothesized that stressful management procedures will exacerbate the putative negative effects of poor housing conditions on resilience. In commercial pig production, many pigs are exposed to regrouping with other unfamiliar pigs, usually accompanied by relocation to a different pen. This mixing of unfamiliar pigs generally leads to vigorous fighting to establish a new dominance hierarchy (15, 16) and is known to be highly stressful to pigs (17, 18).

In this study, we investigated the combined effects of diverging environmental conditions from weaning and allostatic load, created by repeated mixing, on the resilience of pigs. Pigs were either exposed to standard, rather stimulus-poor conditions from weaning onwards or provided with sawdust, extra toys, regular access to a “play arena” and positive human contact (daily brushing), all of which are known to enhance the welfare of pigs (11, 19–21). Half of the pigs were exposed to repeated mixing. To assess their resilience, the response to and recovery from a lipopolysaccharide (LPS) challenge and a Frustration challenge were studied. An LPS injection can induce a temporary sickness response without generating disease (22–24). LPS is an endotoxin that induces an immune reaction (25, 26) and increases oxidative stress (27), leading to oxidative damage (28, 29). We evaluated the response to this challenge by measuring reactive oxygen metabolite (dROM) levels, which reflect oxidative stress (30, 31), and cortisol. As the elevation of cortisol following stressful situations can cause an increase in glucose and lactate (32), these were also assessed, as well as the febrile (fever) response to LPS. In the Frustration challenge, pigs were isolated in a novel pen with a direct view on other pigs playing with enrichment they could not join. Here, we measured standing alert (“freezing”), and escape behavior, both of which have been suggested to indicate a negative emotional state and high stress level (33–36). In addition, ear and tail postures, which are emerging as important indicators of emotional state in domestic pigs (37), and salivary cortisol were assessed.

Apart from the response to these challenges, natural antibody titres, which have been associated with disease resilience and survival in chickens and pigs (4, 38, 39), were measured. Besides, hair cortisol, which is increasingly used as a biomarker for chronic stress (40–42) and resilience (43–45), and growth were measured.

It was hypothesized that the enriched housing and management conditions, as compared with barren conditions, would improve the resilience of pigs, as reflected in a lower response to the challenge and/or a quicker recovery to the pre-challenge state, whereas repeated mixing, expecting to result in high allostatic load, would negatively impact their resilience. Moreover, repeated mixing was expected to exacerbate the negative effects of barren housing on resilience.

## Materials and Methods

The established principles of laboratory animal care and use were followed, as well as the Dutch law on animal experiments. The Animal Care and Use Committee of Wageningen University & Research approved the experiment (DEC code: AVD1040020186245).

### Animal and Housing

In this experiment, 384 female pigs (*Sus scrofa domesticus, TN70* × *Tempo crossbred*) from 65 sows, equally divided over 6 successive batches (*n* = 64 pigs per batch, the sample size was based on power calculations) were studied. We used pigs from one sex only to reduce within-treatment variation. Multiparous sows (parity means ± SEM: 4.9 ± 0.2) were inseminated on the same day within a batch. Piglets were raised on a conventional commercial farm and kept with the sows in farrowing pens until weaning at on average 4 weeks of age (4 weeks in batch 1 and 2, and, due to logistic on-farm issues, 5 weeks in batch 3 and 4, and 3 weeks in batch 5 and 6). A week before weaning, on average at 3 weeks of age, piglets were weighed (B1: 6.4 ± 0.2, B2: 6.9 ± 0.1, B3: 8.9 ± 0.2, B4: 9.4 ± 0.2, B5: 5.0 ± 0.1, B6: 5.1 ± 0.1 kg), and blood and hairs were sampled pre-weaning at their original farm to establish a baseline before receiving any experimental challenge. Pigs were not tail-docked.

After weaning, 380 pigs were transported to Carus, the animal research facility of Wageningen University & Research, Wageningen, the Netherlands. From batch 6, *n* = 60 pigs were used post-weaning as four pigs from other batches had died on-farm and had been replaced by four other pigs. To prevent exceeding the maximum number of pigs allowed to be included in this experiment, this was compensated for batch 6.

All pigs were housed in same sized pens (1.2 × 2.85 m^2^) with 0.85 m^2^ per pig. Pigs were all fed with a standard commercial diet for growing pigs *ad libitum* from a single space pig feeder and each pen had one drinking nipple. The first 3 days after arrival, piglets received a mix of creep feed and weaner diet. The temperature was set at 28°C for the first 6 days, and then at 26°C until the end of the experiment (at an average of 7 weeks of age). One heating lamp was provided in each pen for the first week after weaning. Pigs were given a 12 h day-night regime with 115 Lux from 7:00 until 19:00 h and 30 Lux during the night. The transition between the day and night lighting was done progressively for 10 min. No natural day light was available. A radio was on between 7:00 and 19:00 h.

### Treatments

**Figure 1:**
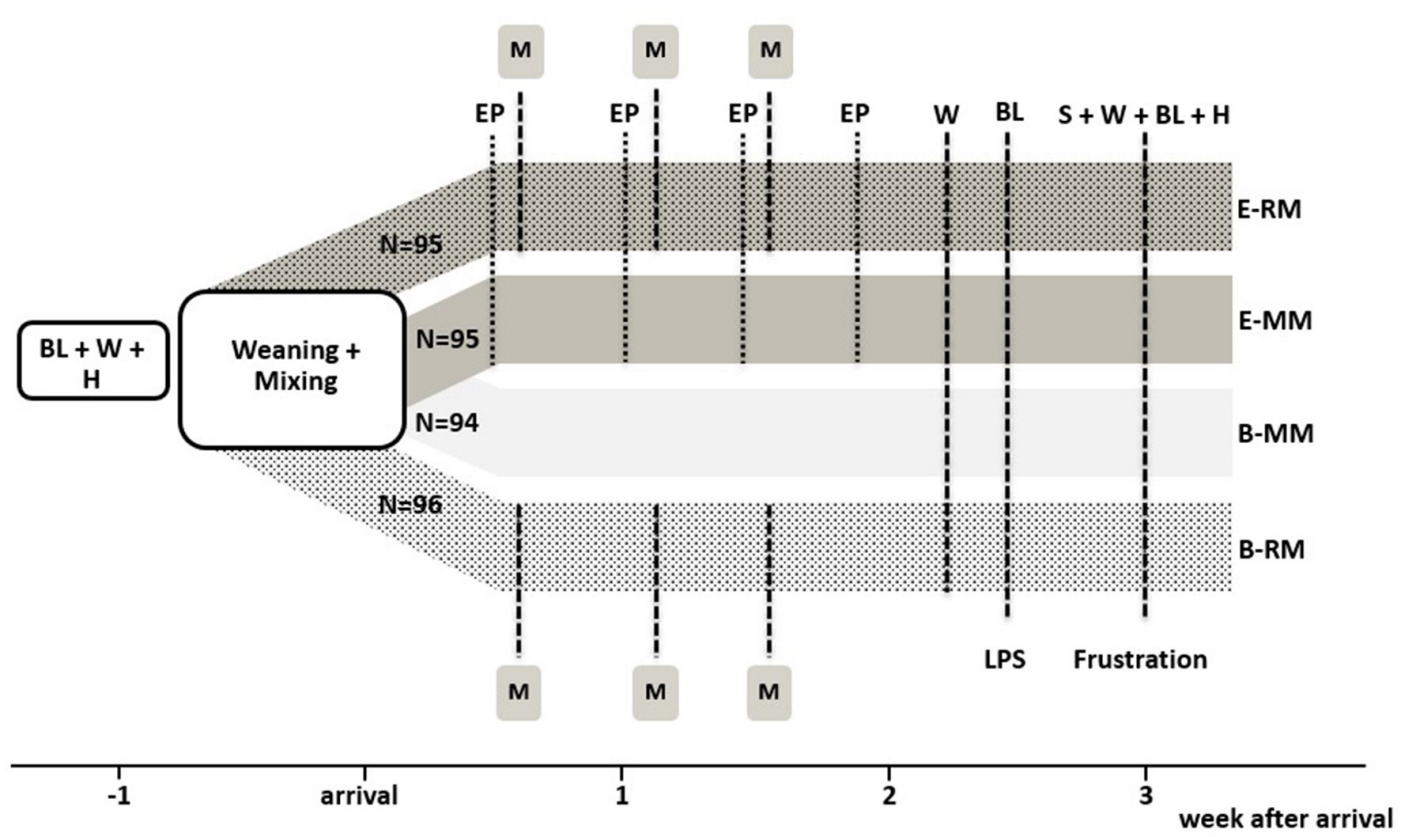
Experimental setup with overview of the four treatment groups and experimental procedures. BL indicates blood sampling, W indicates weighing, H indicates hair sampling, EP indicates 20-min access to the enriched “play arena”, M indicates mixing, S indicates saliva sampling. N refers to the number of pigs per treatment.

After weaning and transport to Carus pigs were subjected to one of four treatment combinations in a 2 × 2 factorial design, with housing and management (enriched, E vs. conventional, B) and mixing (minimal, MM vs. repeated, RM) as factors. All pigs were housed in groups of four unfamiliar pigs. There were *n* = 24 pens per treatment combination (E-RM: 95, E-MM: 95, B-RM: 96, and B-MM: 94 pigs). The experimental set up is shown in Figure 1. Weight and litter were balanced over pens and treatments.

#### Housing and Management Treatment

The E housing and management treatment consisted of enrichment in the form of bedding and extra toys in the home pen, regular access to a ‘play arena” and positive human contacts. The floor of E pens was solid and enriched with sawdust (1 cm layer) and the dirty spots were replenished daily to keep this layer. As well as this, E pigs were provided with extra permanent toys (a jute sack, a brush and a jute rope) and the following toys were swapped over every four days [a chewing ball for dogs (Adori Latex Toy Ball With Squeaker - Dog Toy - 9.5 cm Assorted), a suspended tire dog toy (Kong Toy Traxx Black - Dog Toys), a suspended porcichew® toy, a green MS Schippers Bite cylinder®, a red ring Kong, a blue stick light toy (Adori Led Tpr), a luna pig toy (Easyfix play material Luna 86) or a suspended wooden bar]. Moreover, E pigs were exposed to positive human contacts (each pig was gently brushed for 1 min daily with a soft broom). In addition, E pigs were provided 20 min access with their pen mates to an enriched “play arena” every 3 days for 4 times in total. The enriched “play arena” always contained 20–22 L of peat, a dog agility tunnel (length 200 cm, diameter 40 cm, Trixie) and three microfibre mops attached to a stick floating approximately 40 cm above the ground. On the first and third day of access, also a big yellow ball, a plastic snake, a rope, and two dog toy plastic bones were present, and on the second and fourth day a big white bucket, a rubber ring, a KONG Wubba™ and two pilate balls.

Pigs in the conventional treatment were housed in pens with partly solid (4.4 m^2^) and partly slatted floor (6.8 m^2^). They were provided one chain with bolts and one chain with a porcichew® toy, which were not changed during the experiment.

#### Mixing Treatment

Pigs in half of the B and E pens were regrouped in a new pen with the same housing treatment with three new unfamiliar pen mates every 4 days, on day 0, 4, 8 and 12 after arrival (i.e. they were mixed 4 times, including mixing at weaning), which was expected to create a high allostatic load due to repeated mixing. This group is referred to as RM (repeated mixing). The other half only experienced mixing at weaning and were expected to have a lower allostatic load. This group is referred to as MM (minimal mixing).

#### Observations After Mixing

The frequency of aggressive behaviors (including mounting) and tail and ear biting of RM pigs in their home pen were observed live by four observers after each mixing for 3 h (see ethogram in Table 1) by four observers. If mounting, ear biting or tail biting lasted more than 30 sec, a new count was given. Tail and ear biting were not recorded in batch 1, for the first three mixings. There were six 30-min observation periods for each pen. Observers were balanced over treatments and pens.

**Table 1:** Ethogram of observation after mixings.

**Behavior**	**Definition**
Aggression	Aggressive acts such as head knocking (ramming or pushing pen mate with the head, without biting), biting (ramming or pushing pen mate with the head, with biting), threatening (head movement toward another pig, without a head knock or bite, leading to an avoidance from the pen mate it is dedicated to), chasing (running after a pen mate, usually following another aggressive act), mounting (jumping on top of another pig) or fast succession of mutual aggressive acts (fight).
Ear biting	Biting, chewing or nibbling the ear of a pen mate. Chewing on the ear tag is not included.
Tail biting	Biting, chewing or nibbling the tail of a pen mate.

### Challenges

Resilience of the pigs in the four treatment groups (E-RM, E-MM, B-RM, and B-MM) was assessed by following the response to and recovery process after an LPS injection to induce a sickness response, and to a Frustration challenge to induce a social/psychological stress response.

#### LPS Challenge

Two weeks after arrival at the experimental farm, 372 pigs (6 pigs were not included due to health issues around the challenge and two pigs were euthanized because they had health problems) were intravenously injected via a catheter (this catheter was removed immediately after injection) in the hind leg with 2 μg of LPS/kg of body weight (LPS sigma L4391 *Escherichia coli* O111:B4) to induce an acute systemic inflammatory response, without generating disease as previously described (24). Due to time constraints, the challenge was carried out on two consecutive days in each batch, balanced for treatments, i.e. half of the pens from each treatment group were tested on one day. Blood samples (10 ml) were collected by jugular vein puncture in both EDTA tubes (Greiner Bio-one GmbH, Austria) and in serum tubes just before the LPS injection at 0 h (baseline) and at 1, 3, and 5 h after the LPS injection. At these time points, rectal temperatures were also measured (before each blood sampling to avoid an artificial increase due to handling).

#### Frustration Challenge

Three weeks after arrival at the experimental farm, pigs were exposed to the Frustration challenge. A total of 373 pigs were tested, as one pig died due to a health issue and four pigs had been euthanized (one pig with poor growth and lack of appetite, one pig due to health issues and two pigs because they poorly recovered from the LPS challenge. Post mortem investigation revealed an infection which may have caused the unexpected detrimental response to the LPS challenge). Each pig was separated from the home pen and moved to an individual novel pen (1.2 × 0.6 m) in the room of the “play arena” for 10 min with a direct view on four unfamiliar pigs from another pen, moving and playing freely in the “play arena” as part of their enrichment treatment. The inability to join the other pigs may induce frustration. Saliva samples were taken 5 times [a baseline for all pigs at 6:30 h in their home pens, before the challenge started (baseline at a fixed time), at −5 min (prior to the start of the challenge in a cart) and at 20, 40, and 60 min after the start of the challenge in their home pens]. Saliva samples were collected with Salivettes® containing polypropylene swabs (Sarstedt Inc 51.1534.500). The pigs, who were habituated to the procedure before the challenge, were allowed to chew for 1–2 min on the swabs which were held by clamp forceps. Behavior, tail and ear postures, and vocalizations (see ethogram in Table 2) of the pigs were recorded live by two observers during the challenge using continuous behavior recording. One of the observers scored the behavior and vocalizations of the tested pig, and the other the ear and tail postures. For those observations, tablets with the Observer 14.2 software (Noldus Information Technology, Wageningen, The Netherlands) were used.

**Table 2:** Ethogram for the Frustration challenge.

**Behavior**	**Definition**
***Behavior class***	
Standing alert	Standing motionless with head fixed (up or down) and ears upright
Escaping	Jumping against the wall of the test pen
Excreting^a^	Defecating and urinating
Other behavior	Initial state, the pig is not performing the behaviors mentioned above
***Vocalizations***	
Grunt^a^	Vocalization with a low tone
High-pitched vocalization^a^	Vocalization that contains a high tone, such as a squeal or scream
Bark^a^	Vocalization with a low tone that sounds like “wuff”
***Ear posture class***	
Ear forward	Both ears directed forward
Ear backward	Both ears directed backward
Ear mix	One ear directed forward and one ear backward
***Tail posture class***	
Tail in curl	Raised tail forming a loop
Tail wagging	Tail swings in any direction, but mostly from side to side
Tail hanging	Low straight or low hanging tail
Tail tucked	Tail tucked between legs

a *scored as events, all other behaviors as states*.

### Blood and Saliva Analysis

The EDTA tubes were centrifuged at 1000 g for 20 min at 4°C and plasma samples were stored at −20°C until analysis. Serum tubes (Greiner Bio-one, Alphen aan den Rijn, the Netherlands) were kept at ambient temperature for at least 30 min and centrifuged at 1,500 g for 10 min at 4°C and serum samples were also stored at −20°C until analysis. The Salivettes® with the saliva samples were centrifuged at 1,500 g for 10 min at 4°C. Saliva samples were stored at −20°C until laboratory analyses.

Cortisol in EDTA plasma samples was assessed using the cortisol RIA kit from Immunotech (Beckman-Coulter, ref IM1841, Czech Republic). Serum concentrations of glucose, lactate and reactive oxygen metabolites (dROM) were determined using commercial kits (Glucose: HK 981304, Thermo Fisher Scientific, Courtaboeuf, France; Lactate: A11A01721, Horiba ABX SAS, Montpellier, France; dROM: test MC002, Diacron Labs S.R.L., Grossetto, Italy) which were developed for a clinical chemistry analyser Konelab 20i (Thermo Fisher Scientific, Courtaboeuf, France).

Salivary cortisol was measured using the cortisol kit (Enzyme immunoassay for the quantitative determination of free cortisol in human saliva, ref RE52611) from IBL International GmbH (Hamburg, Germany).

Blood serum samples taken at 3 (on commercial farm), 6 (before LPS injection) and 7 weeks of age (1h after the Frustration challenge) were used to detect natural antibody levels. Titers of IgM and IgG in serum binding keyhole limpet hemocyanin (KLH) and phosphoryl choline-conjugated to Bovine Serum Albumin (PC-BSA) were measured as previously described by Luo et al. (46), except for the coating concentration for PC-BSA, which was 1 μg/ml in this study.

### Body Weights

Pigs were weighed at on average 3 (on commercial farm), 6 (before the challenges) and 7 weeks of age (1h after the Frustration challenge). Relative growth was estimated as (current weight – initial weight)/(initial weight) × 100. Initial weight was the weight at the start of the period over which growth was measured, e.g. for relative growth between week 3 and 6 and between week 6 and 7, these were the weights of week 3 and 6, respectively.

### Hair Samples

Hair samples were collected at on average 3 weeks of age, and 7 weeks of age, at the end of the experiment. The shaving area of about 15 cm^2^ was realized on the same body part for all piglets to avoid potential bias related to location. The location was close to the hip of the pigs, at the connection between the abdominal area and the hind leg. A single-use surgical razor was used for each pig. Samples were collected, wearing gloves to avoid any contamination of the samples. At week 3, only the right side of the pigs was shaved, while it was the left side at week 7. Samples were stored in aluminum foils at room temperature in the dark until analysis.

Hairs (112 samples × 2 times from batch 1 and 2, *n* = 28 pigs per treatment) were prepared following the same protocol as described in Parois et al. (submitted) and cortisol concentration was determined using the high sensitivity salivary cortisol ELISA kit (ref 1-3002) from Salimetrics (Pennsylvania, USA).

### Statistical Analyses

Statistical analyses were performed with SAS (SAS 9.4, SAS Institute Inc.). Three pigs were excluded from the LPS challenge analysis as they mistakenly received an incorrect dose of LPS.

#### Behavior After Mixing

Behaviors after mixing were analyzed using a linear mixed model with enrichment (enriched vs. barren), mixings (mixing 1, 2, 3, 4) and their interaction as fixed effects and litter (nested within batch), batch, and pig (nested within enrichment, batch and litter) as random effects.

#### Measurements in Blood and Saliva in the LPS and Frustration Challenge

Blood levels of cortisol, glucose, lactate, and dROM, salivary cortisol and rectal temperature measured around the challenges were analyzed using a repeated linear mixed model with an autoregressive (1) covariance structure (repeated effect with pig as subject). The fixed effects of enrichment (E vs. B), mixing (RM vs. MM), sampling time and their interactions and the random effects of litter, batch, pen and pen within time were included. Levels of lactate, dROM and salivary cortisol were log transformed to obtain normality of residuals. To account for potential time of day effects, starting time of the LPS challenge (relative to the first pig on that day) was added as a covariate to the model for the analysis of blood variables and rectal temperature. It only affected glucose levels, with slightly higher levels for pigs tested later on a day and was omitted from the models for the other variables. In the initial model for salivary cortisol, the baseline sample at 6:30 was also included. Preliminary analysis, however, revealed that this did not differ from the sample at *t* = 0, and therefore it was removed from the final analysis.

Areas under the recovery curves (AUC) of the blood and saliva variables and rectal temperature were approximated from repeated measurements using the trapezoidal rule. AUC were analyzed using a linear mixed model with enrichment, mixing and their interaction as fixed effects and litter (nested within batch), batch, and pen (nested within enrichment, mixing and batch) as random effects.

#### Behavior During the Frustration Challenge

Frequencies of standing alert, escape behavior and other behavior were summed and referred to as the “frequency of behavioral transitions”. Behavioral variables were analyzed using the same mixed models as used for the AUC. Duration of standing alert was log transformed and number of high-pitched vocalizations and frequency of excreting were square root transformed to obtain normality of residuals. As only a small number of pigs (*n* = 73 pigs) showed a tucked tail, this behavior was changed into a binary variable (0 = tucked, 1 = not tucked), which was analyzed by a generalized linear mixed model with binary distribution and logit link function, with the same fixed and random effects as for the other behaviors. Only 11 pigs showed tail wagging and barking never occurred, so these variables were not included in the analysis.

#### General Measurements

Cortisol, glucose, lactate and dROM measured at the end of the experiment (1h after the Frustration challenge) were analyzed using the same mixed model as for the AUC. Lactate and dROM levels were log transformed to obtain normality of residuals.

Natural antibody titers were analyzed using a repeated linear mixed model [autoregressive (1) structure], with fixed effects of enrichment, mixing, week, and their interactions and random effects of litter (nested within batch), batch and pig (nested within enrichment, mixing and batch). Titers of KLH-IgM and KLH-IgG were log transformed to obtain normality of residuals.

Cortisol concentration in hair samples were analyzed by the same model as used for the natural antibody titers.

Relative growths were analyzed using a linear mixed model with enrichment, mixing and their interaction as fixed effects, and litter (nested within batch) and batch as random effect. Relative growth from week 6 to week 7 was square root transformed to obtain normality of residuals.

Significant interactions (*p* < 0.05) were further investigated with *post-hoc* pairwise comparisons using the difference of the least square means. If interactions with time were found, pairwise comparisons were adjusted using Tukey corrections. Results are presented as means ± SEM.

## Results

### Mixing Observations

**Figure 2:**
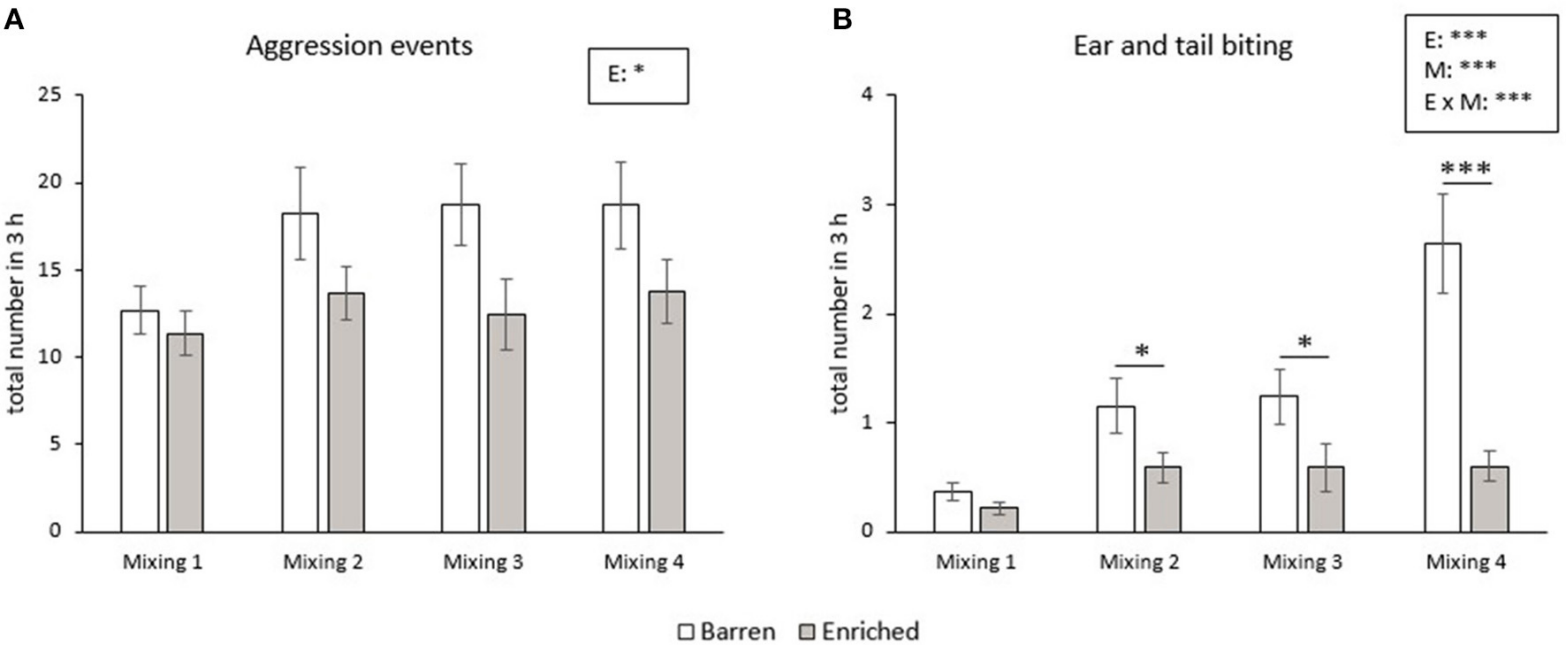
Means and SEM of frequencies of aggression **(A)** and ear and tail biting **(B)** during the 3 h after each mixing in pigs exposed to a barren or enriched treatment. Significant effects of enrichment (E), mixings (M: mixing 1, 2, 3, 4), their interactions are indicated: *P < 0.05, ***P < 0.001.

The number of aggressive events did not change from the first until the fourth mixing in the RM groups. B pigs showed more aggressive events during the 3 h after mixing than E pigs (*p* = 0.011, Figure 2A). The frequency of ear and tail biting was affected by enrichment (*p* < 0.001), mixing (*p* < 0.001) and their interaction (*p* < 0.001). The frequency of ear and tail biting increased from the first to the fourth mixing in B pigs (all *p* < 0.001), but did not significantly change over time in E pigs. In addition, B pigs had a higher frequency of tail and ear biting in the second, third and fourth mixing than E pigs (all *p* < 0.05, Figure 2B).

### LPS Challenge

**Figure 3:**
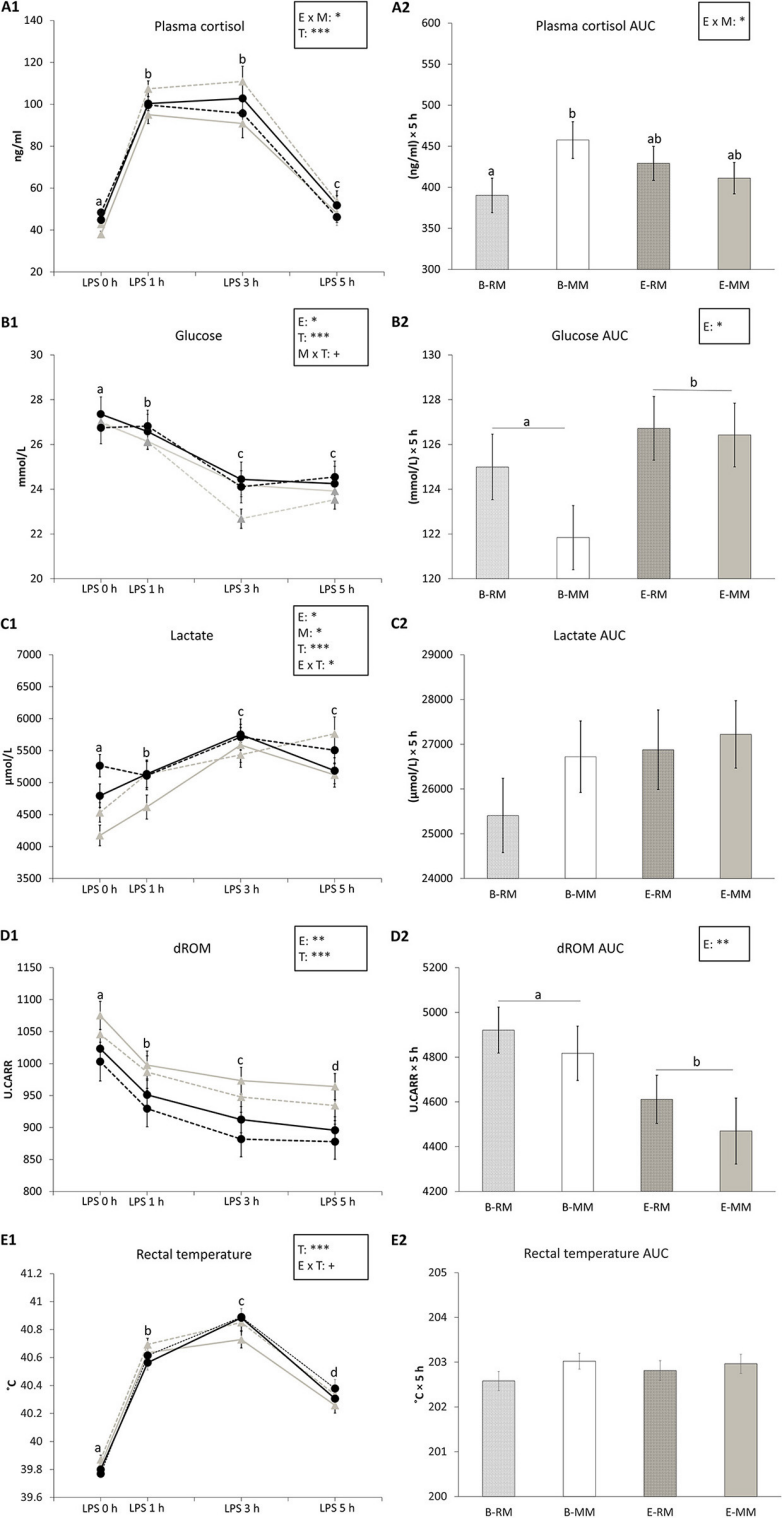
The means and area under the curve (AUC) with SEM of cortisol, glucose, lactate and dROM levels in blood and rectal temperatures measured after an LPS challenge in pigs which were exposed to either a barren (B) or an enriched (E) treatment with repeated mixings (RM) or minimal mixing (MM). **(A1–E1)** show the means and SEM of four treatment groups over time. **(A2–E2)** show the means and SEM of the AUC in each treatment group. Groups and sampling times lacking a common letter (a, b, c, d) significantly differ. Significant effects of enrichment (E), mixing (M), sampling time (T) and their interactions are indicated: ***p < 0.001, *p < 0.05, ^+^*p* < 0.10.

Plasma cortisol and its area under the curve (AUC) were affected by the enrichment × mixing interaction (*p* = 0.036 and *p* = 0.041, respectively) with higher values for B-MM pigs than B-RM pigs in both and levels of E pigs in between (Figures 3A1,A2). Plasma cortisol levels were also affected by time (*p* < 0.001), with higher levels at 1 h and 3 h after LPS injection than before the challenge and after 5 h, and higher levels at 5 h than before the LPS injection (Figure 3A1).

Glucose levels in serum were affected by enrichment (B: 25.1 ± 0.1, E: 25.6 ± 0.1 mmol/L, *p* = 0.046, Figure 3B1), time (*p* < 0.001) and tended to be affected by the mixing × time (*p* = 0.085) interaction. Glucose levels decreased after LPS injection and remained at a lower level at 3 h and 5 h after LPS injection (Figure 3B1). Glucose AUC was only affected by enrichment, with lower values for B pigs than E pigs (B: 123.2 ± 1.0, E: 126.6 ± 1.0 (mmol/L) × 5 h, *p* = 0.028, Figure 3B2).

Lactate levels in serum were affected by enrichment (*p* = 0.015, Figure 3C1), time (*p* < 0.001) and enrichment × time (*p* = 0.037). Pairwise comparison showed that just before LPS injection, E pigs had higher levels of lactate than B pigs (*p* = 0.007), but at other sampling times, there was no difference (Figure 3C1). Lactate level at 3 h after LPS injection was higher than the levels at 0 and 1 h in E pigs (all *p* < 0.05) and lactate levels at 3 and 5 h were higher than the levels at 0 and 1 h in B pigs (all *p* < 0.05). Lactate levels were also affected by mixing, with higher levels in MM pigs than RM pigs (MM: 5304.55 ± 73.86, RM: 5045.8 ± 76.03 μmol/L, *p* = 0.031, Figure 3C1). There was no effect of enrichment or mixing on lactate AUC (Figure 3C2).

dROM levels in serum and dROM AUC were both affected by enrichment (*p* = 0.006 and *p* = 0.008, respectively) with higher values for B pigs than E pigs (B: 990.7 ± 8.3, E: 934.8 ± 9.1 U.CARR, Figure 3D1; B: 4,868.9 ± 79.4, E: 4,541 ± 90.7 U.CARR × 5 h, Figure 3D2). dROM levels were also affected by time (*p* < 0.001) with decreasing levels in serum after LPS injection (Figure 3D1).

Rectal temperatures were affected by time (*p* < 0.001) and tended to be affected by enrichment × time (*p* = 0.057), without significant pairwise differences. Pigs' temperature increased after LPS injection and reached a peak at 3 h after LPS injection (Figure 3E1). Rectal temperature AUC was not affected by any factor (Figure 3E2).

### Frustration Challenge

#### Salivary Cortisol

**Figure 4:**
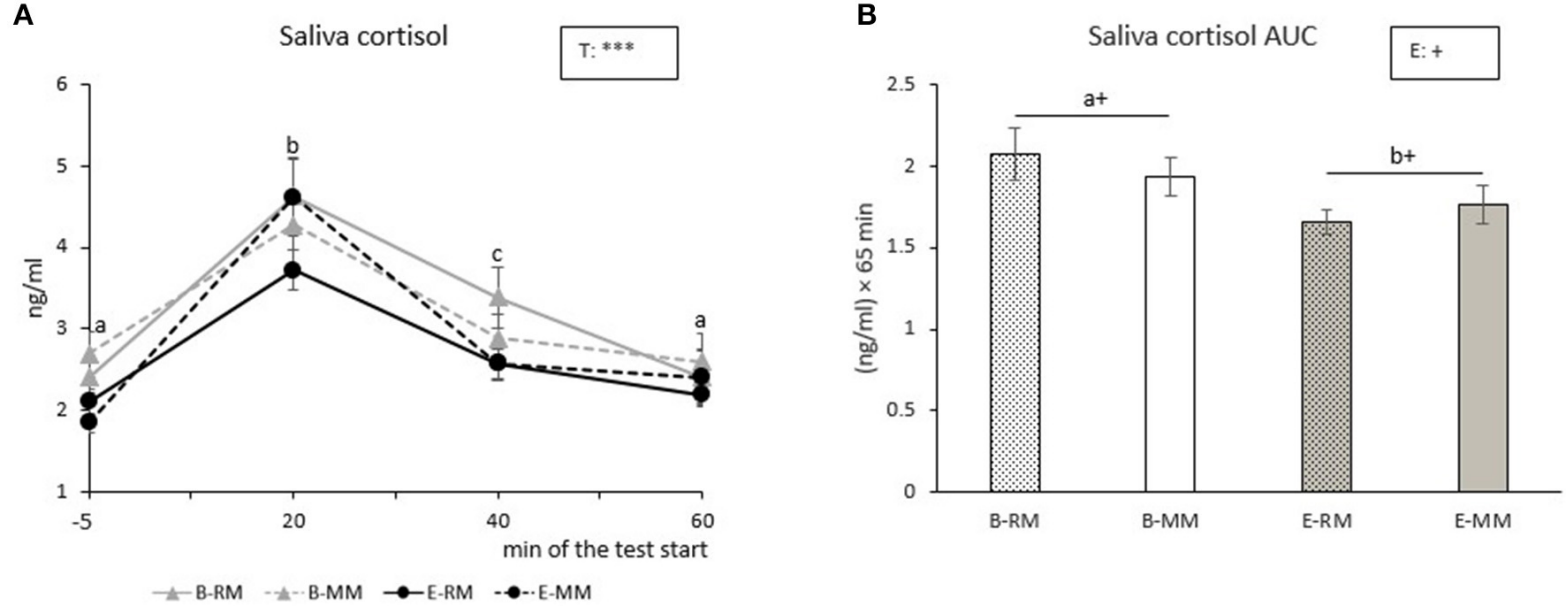
The means of cortisol levels over sampling times **(A)** and area under the curve (AUC) with SEM **(B)** after a Frustration challenge in saliva samples taken from pigs which were exposed to either a barren **(B)** or an enriched (E) treatment with repeated mixings (RM) or minimal mixing (MM). Groups and time lacking a common letter (a,b,c) significantly differ and + shows a tendency in difference. Significant effects of enrichment (E) and sampling time (T) are indicated: ***p < 0.001, ^+^*p* < 0.10.

Salivary cortisol levels were only affected by time (*p* < 0.001). *Post-hoc* pairwise comparisons showed that saliva cortisol levels increased with a peak at 20 min after the Frustration challenge, and then the levels decreased until they reached the original basal level at 60 min after the start of the challenge (Figure 4A). Saliva cortisol AUC tended to be affected by enrichment with higher values for B pigs than E pigs [B: 2.0 ± 0.1, E: 1.7 ± 0.1 (ng/ml) × 60 min, *p* = 0.096, Figure 4B].

#### Behaviors, Vocalizations, Ear and Tail Postures

Table 3 shows the behaviors, vocalizations, ear and tail postures of the pigs during the Frustration challenge and the significances of the effects of enrichment, mixing and their interaction. B pigs spent more time on standing alert and on escape behavior than E pigs (*p* = 0.024 and *p* = 0.003, respectively). B pigs excreted less often than E pigs (*p* = 0.002) and changed their behavior more often (frequency of behavior transitions) than E pigs (*p* < 0.001).

**Table 3:** Behaviors, vocalizations, and ear and tail postures of pigs which were exposed to either a barren (B) or an enriched (E) treatment with repeated mixings (RM) or minimal mixing (MM), recorded in a 10 min Frustration test.

**Variables**	**B-RM**	**B-MM**	**E-RM**	**E-MM**	**E**	**M**	**E × M**
***Behavior***
Standing alert (sec)	17.6 ± 2.7	16.4 ± 2.6	8.2 ± 1.2	11.6 ± 1.9	^*^	ns	ns
Escape (sec)	229.0 ± 11.0	231.4 ± 10.9	205.0 ± 10.9	189. ± 10.7	^**^	ns	ns
Excrete (N)	3.2 ± 0.3	3.0 ± 0.2	3.9 ± 0.3	3.5 ± 0.2	^**^	ns	ns
Behavioral transitions (N)	21.0 ± 0.9	21.2 ± 0.9	17.9 ± 0.7	18.2 ± 1.0	^***^	ns	ns
***Vocalizations***
Grunt (N)	104.2 ± 5.5	104.6 ± 5.9	107.4 ± 5.1	92.7 ± 5.6	ns	+	ns
High-pitched (N)	43.0 ± 4.8	51.8 ± 5.3	52.9 ± 5.5	41.3 ± 5.0	ns	ns	^*^
***Ear and tail postures***
Ear forward (sec)	426.2 ± 14.1	468.9 ± 12.2	386.4 ± 13.9	393.0 ± 14.6	^***^	+	ns
Ear backward (sec)	17.2 ± 4.1	14.0 ± 3.6	27.0 ± 5.6	27.7 ± 4.8	^*^	ns	ns
Ear mix (sec)	156.6 ± 13.0	117.3 ± 11.2	186.4 ± 11.7	179.3 ± 12.7	^***^	^*^	ns
Tail in curl (sec)	438.4 ± 18.4	474.1 ± 16.6	511.8 ± 14.2	500.9 ± 16.3	^***^	ns	ns
Tail hanging (sec)	124.5 ± 15.5	101.1 ± 14.1	81.2 ± 13.7	90.7 ± 15.6	^***^	ns	ns
Tail tucked (N of pigs)	27	24	11	11	^**^	ns	ns

*E indicates the effect of enrichment (barren vs. enriched), M indicates the effect of mixing (repeated vs. minimal), and E × M indicates the interaction effect between enrichment and mixing. Significances of differences are indicated*:

*** *p <0.001*,

** *p <0.01*,

* *p <0.05, and ^+^p <0.10; ns indicates non-significance. N indicates the frequency*.

High-pitched vocalizations were affected by the enrichment × mixing interaction (*p* = 0.043). *Post-hoc* pairwise comparisons revealed that E-MM pigs tended to utter less high-pitched vocalizations than E-RM (*p* = 0.094) and B-MM pigs (*p* = 0.087), with B-RM in between. Grunts tended to be affected by mixing (*p* = 0.089), with RM pigs tending to utter more grunts than MM pigs.

The direction of pigs' ears during the test was more forward than backward or mixed. B pigs spent more time with ears forward than E pigs (enrichment effect, *p* < 0.001). In addition, the RM pigs tended to show less ear forward than MM pigs (mixing effect, *p* = 0.059). Conversely, time spent with one ear backward and one forward, i.e. ear mix, was higher in E pigs than in B pigs (*p* < 0.001) and higher in RM pigs than in MM pigs (*p* = 0.042). Moreover, B pigs spent less time with ears backward than E pigs (*p* = 0.028).

Pigs' tails were in a curl for most of the time, and very few pigs wagged their tails. The time of tail-in-curl was affected by enrichment only, with less time in B pigs than in E pigs (*p* < 0.001). The time of tail hanging was affected by enrichment as well and was longer in B pigs than in E pigs (*p* < 0.001). Additionally, a higher number of B pigs than E pigs showed a tucked tail (*p* = 0.003).

#### Metabolic Parameters and Plasma Cortisol at the End of Experiment

There was no effect of enrichment or mixing on glucose and lactate levels in the blood sample taken 1 h after the Frustration challenge. Enrichment tended to affect dROM levels (B: 981.94 ± 16.31, E: 946.17 ± 16.42 U.CARR, *p* = 0.063). The cortisol level in plasma taken 1 h after the Frustration challenge was affected by housing treatment with higher levels for B pigs than E pigs (B: 29.06 ± 1.10, E: 25.32 ± 0.91 ng/ml, *p* = 0.001).

### Natural Antibody Binding KLH and PC-BSA

**Figure 5:**
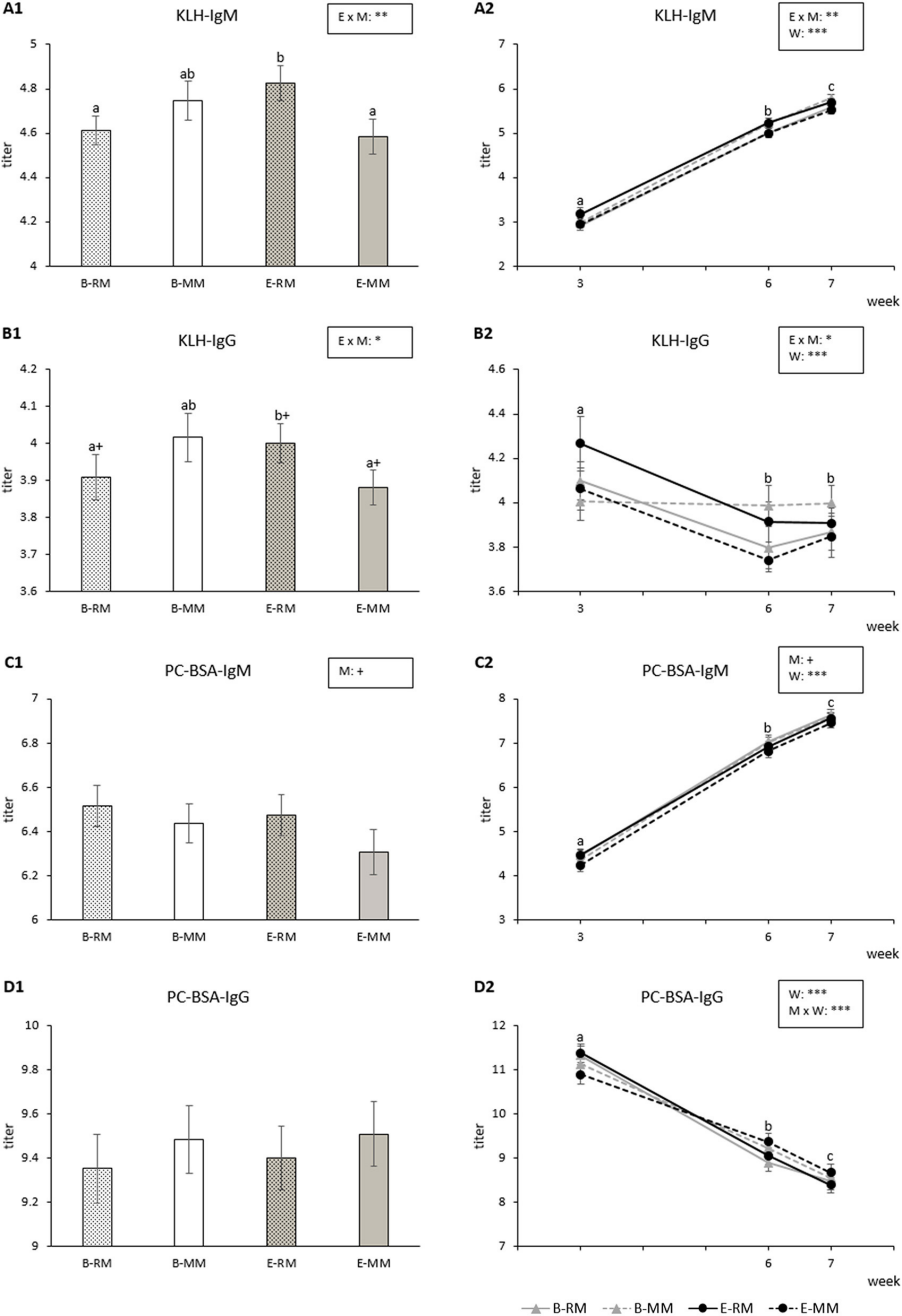
Means and SEM of the natural IgM and IgG binding KLH and PC-BSA titers over three sampling times in pigs which were exposed to either a barren (B) or an enriched (E) treatment with repeated mixings (RM) or minimal mixing (MM). **(A1–D1)** show the average of three sampling times and **(A2–D2)** show the average of each sampling time. Groups and time lacking a common letter (a,b,c) significantly differ and + shows a tendency in difference. Significant effects of enrichment (E), mixing (M), week (W) and their interactions are indicated ***p < 0.001, **p < 0.01, *p < 0.05, ^+^*p* < 0.10.

KLH-IgM titers were affected by the enrichment × mixing interaction (*p* = 0.009) with higher titers for E-RM pigs than for B-RM and E-MM pigs (Figure 5A1). KLH-IgM titers were also affected by week (*p* < 0.001) and generally increased over weeks for all groups (Figure 5A2).

KLH-IgG titers were also affected by the enrichment × mixing interactions (*p* = 0.023). Pairwise comparisons revealed that titers tended to be higher for E-RM pigs than for B-RM (*p* = 0.092) and E-MM pigs (*p* = 0.052, Figure 5B1). KLH-IgG titers were affected by week (*p* < 0.001), with higher levels in week 3 than in week 6 or 7 (*p* < 0.001, Figure 5B2).

PC-BSA-IgM titers were affected by week (*p* < 0.001) with increasing titers over weeks for all groups (Figure 5C2). RM pigs tended to have higher titers than MM pigs (*p* = 0.050, Figure 5C1).

PC-BSA-IgG titers were affected by week (*p* < 0.001), with a general decrease over time, and by the mixing × week interaction (*p* = 0.002; Figure 5D2), without significant pairwise differences (Figure 5D1).

### Growth

Relative growth before the LPS and Frustration challenges, between week 3 and 6, was not affected by enrichment or mixing. RM pigs gained relatively more body weight from week 6 to week 7 (week of the LPS and Frustration challenges) than MM pigs (RM: 32.7 ± 0.6, MM: 30.2 ± 0.6%, *p* = 0.014) and more weight over the whole experimental period (RM: 133.0 ± 2.1, MM: 125.0 ± 2.3%, *p* = 0.004). No enrichment effect was found on relative growth.

### Hair Cortisol Concentration

Hair cortisol concentration was not affected by enrichment or mixing treatment, but the concentration was lower in week 7 (24.0 ± 0.7 pg/mg of hair) than in week 3 (31.4 ± 1.2 pg/mg of hair, *p* < 0.001).

## Discussion

The effects of an enriched housing and management treatment, and repeated mixing on resilience in pigs were assessed in this study. Enriched pigs were expected to show enhanced resilience as compared with pigs kept in a barren environment. Repeated mixing was expected to result in a high allostatic load, and therefore a reduced resilience to challenges, which could be exacerbated in barren pigs. Although we found some indications of more favorable responses to the challenges in enriched pigs, repeated mixing did not seem to reduce resilience. Contrary to expectations, repeatedly mixed pigs gained relatively more weight than pigs mixed only once during the experiment, especially in the week of the challenges. Moreover, the repeatedly mixed barren pigs showed a lower cortisol response in the LPS challenge than their counterparts that were only mixed once.

### Enrichment Effects

The enriched pigs were exposed to positive human contact (1 min of stroking with a brush per day), sawdust bedding and toys, and regular access to a “play arena”. These conditions all have been demonstrated to improve welfare of pigs (11, 19, 21), and, moreover, different types of enrichment have been shown to enhance their resilience (10). Enrichment in this study did, however, not improve the speed of recovery from the LPS and Frustration challenges in pigs overall, but influenced the response of some variables to these challenges.

Several studies suggest that levels of glucose and lactate can reflect stress (47, 48), as the elevation of cortisol following stressful situations can cause an increase in glucose and lactate (32). Glucose concentrations decreased in 5 h after LPS injection, which is consistent with a previous study (Parois et al., under review). We do not know why blood glucose levels decreased rather than increased following the LPS challenge in this and the previous study. Possibly, the LPS challenge caused metabolic stress, whereby glucose was taken up from the blood rapidly as the febrile and immune responses following LPS injection are energetically demanding and increase glucose requirements (49, 50). Enriched pigs had higher concentrations and a higher AUC of glucose than barren pigs after LPS injection, which might suggest that enriched pigs were less affected by this challenge. In line with this, it was shown that pigs exposed to environmental enrichment in the form of aromatized bottles showed higher glucose concentrations in plasma within 90 min after weaning stress compare to control pigs Yanez-Pizana et al. (51). The enrichment × time interaction reflected a higher lactate level of enriched pigs before the LPS challenge. This is in contrast with another study which showed lower lactate concentrations in pigs exposed to enrichment in the form of straw bedding and extra space (52). Peak levels of lactate following the LPS challenge did, however, not differ from those of barren pigs, which might suggest that enriched pigs had a smaller increase in lactate. Additionally, levels of lactate were sooner back to basal levels in enriched pigs.

An interaction between enrichment and mixing was found for the cortisol response to the LPS challenge with an impact of mixing on barren pigs only (discussed in Section Mixing Effects and Their Interaction With Enrichment). Barren pigs did, however, not show overall higher cortisol levels following LPS injection than enriched pigs, although a trend for a larger AUC in salivary cortisol following the Frustration challenge was found, as well as a higher plasma cortisol level at 1 h after this latter challenge. The lack of large effects of enrichment on the cortisol response to the LPS challenge is in contrast to another study comparing pigs from an alternative housing system with enrichment to barren housed pigs (Parois et al., under review). In this previous study, which generally revealed more clear effects of enrichment on resilience, pigs were exposed to environmental enrichment from birth onwards, whereas in this study treatments were only applied after weaning. Moreover, both the pre-weaning (group housing with multiple litters) and post-weaning environment (extra space, straw, peat, sawdust, and extra toys) in this previous study diverged more from the barren conditions, which may explain the lack of an enrichment effect in our study. Early life enrichment has a large impact on the welfare of pigs (53–56), especially if it is combined with group housing during lactation (57–59). In pigs, effects of the early life environment on the hypothalamic pituitary adrenal axis may be long-lasting, as it has been shown that piglets housed in a barren environment before weaning, as opposed to piglets provided with bedding material, showed a blunted secretion rhythm in cortisol at 21 weeks of age (53). The late onset of our enrichment treatment might also explain why we did not find an enrichment effect on hair cortisol, in contrast with the study of Parois et al. (under review). In another study, however, pigs housed in barren conditions showed significantly higher hair cortisol concentrations compared with pigs kept in pens with sawdust, natural hemp ropes and rubber ball even though they were raised in similar pens (60). Even though hair cortisol has been advocated as a promising indicator of chronic stress (40, 42, 43, 61, 62), a recent study demonstrated that long-term contamination of hairs with urine causes incorporation of cortisol in the hair shaft, leading to higher accumulation of cortisol in hairs (61, 63). We cannot exclude that the hair cortisol measurements in our study partly reflect a potential difference in exposure to urine in our contrasting environments (partly slatted floor in the barren pens vs. solid floor with sawdust in the enriched pens), which could obscure effects of the enrichment treatment itself. The same might hold for previous studies on housing effects on hair cortisol (64).

Levels of dROM are used to evaluate overall oxidative stress as they reflect hydroperoxides created during peroxidation of amino acids, lipids and proteins (30, 31). The concentration of dROM in plasma increased 24 h after injection with LPS in bats (65), likely as a consequence of physiological processes involved in sickness behavior (66), such as an elevated metabolic rate (67). In our study, we found that the concentrations of dROM decreased after injection of LPS, which were measured within 5 h and may not be comparable to the concentrations measured after 24 h. It is not clear why the concentration of dROM decreased over 5 h after LPS injection. During the first h of the LPS response, there were large blood composition changes, such as a glucose drop, an increase in lactate and changes in other metabolites. Some of the metabolites with a large transitory change in blood concentration during the LPS response, might have influenced the measurement of dROM. In order to see pro-oxidative effects due to LPS, it may be needed to wait until these metabolites are back to baseline concentrations, which was, in our study, not the case for glucose and lactate at 5 h after the challenge. In this study, enriched pigs had lower concentrations of dROM than barren pigs around the LPS challenge and after the Frustration challenge. This is in line with the study of Merlot et al. (68) reporting lower oxidative stress in sows kept in more spacious, straw-bedded pens than in those kept in barren pens. In rodents environmental enrichment has been found to reduce oxidative damage as well (69, 70). Chronic stress caused by barren housing may induce a mild inflammation (71), which would be expressed in higher haptoglobin levels in barren housed pigs (72) and it has been shown that inflammation can increase oxidative stress (73), but more studies are needed to support this.

During the Frustration test, barren pigs seemed to be more stressed than enriched pigs, as barren pigs spent more time on standing alert and escape behavior (33–36). In support of this, barren pigs showed higher frequencies of behavioral transitions, likely reflecting restlessness, displayed less tail-in-curl, showed a hanging tail more often and were more likely to have their tail tucked during the test. A curled tail has been mentioned as a marker of a positive emotional state and good welfare (37, 74). In support of this, curled tails were more often observed in enriched pens compared to barren pens in a recent study (75). Reversely, a tucked tail is associated with stress and fear (76, 77), and is frequently seen before a tail biting outbreak (75, 78, 79). A hanging tail is either seen as a neutral posture or as an indicator of a negative emotional state (36, 80, 81). Therefore, based on the above findings, barren pigs seemed to be more stressed in the test, which was also supported by a tendency for a larger AUC of salivary cortisol than enriched pigs and higher plasma cortisol levels 1 h after the challenge. However, we also found differences that do not point to a higher stress level in barren pigs. Enriched pigs defecated/urinated more often and kept their ears more in a backward position, which could suggest that these pigs had a less positive emotional state than barren pigs during the test (35, 82, 83). It could be that the Frustration test led to different responses in barren and enriched pigs. Although likely all pigs were stressed by being restrained in a small pen, barren pigs may have been more affected by the novelty of the test room, whereas enriched pigs were possibly more frustrated as they knew the “play arena”, but were not able to access it themselves. It should be noted that it cannot be excluded that the cortisol response was, apart from the novel and frustrative situation, partly induced by transportation to and from the test pen. This test has not been used before or validated. We assumed that the tested pigs would be frustrated by not being able to play, but we cannot rule out that the test induced another state in the pigs.

### Mixing Effects and Their Interaction With Enrichment

Repeated mixing did not seem to reduce resilience, and moreover, repeatedly mixed barren pigs showed a lower cortisol response to the LPS challenge than their minimally mixed barren counterparts, suggesting that repeatedly mixed pigs responded less detrimentally than minimally mixed pigs in barren pens. Additionally, repeatedly mixed pigs gained relatively more weight, particularly in the week of the challenges, than pigs that had been mixed only once.

Mixing, i.e. regrouping of unfamiliar pigs, is a highly stressful event for pigs usually leading to vigorous fighting to establish a new hierarchy (17, 18). It has been shown that repeated mixing may lead to chronic stress, as reflected in higher salivary cortisol levels (84) and higher accumulation of cortisol in hairs as compared with non-mixed pigs (64), as well as in long-term changes in blood immune cells (85). We therefore expected the repeated mixing treatment to cause a high allostatic load. This burden of cumulative stress was expected to negatively affect the resilience of the pigs to the challenges and impact long-term indicators of stress. Contrary to expectations, and in contrast with previous papers reporting negative effects of mixing on growth (18, 86, 87), repeated mixing increased post-weaning growth, especially during the week in which the LPS challenge took place. Repeated mixing did not influence the physiological responses to the LPS challenge, except that it affected the plasma cortisol response in an enrichment-dependent manner, with lower levels for repeatedly mixed pigs as compared with minimally mixed pigs in barren pens. Behavioral and salivary cortisol responses to the Frustration test were not different for repeatedly or minimally mixed pigs. There are several possible explanations for the lack of a strong effect of repeated mixing.

First, the contrast with the minimally mixed group may not have been large enough, as this group also had to cope with one regrouping event. Several studies reporting negative effects of mixing on growth, feed efficiency, behavior and cortisol level, compared regrouped pigs with never mixed groups, i.e. pigs kept with their siblings (18, 84, 87). Second, pigs may have been habituated to mixing as they were repeatedly exposed to the same procedure, potentially adapting their behavioral strategy. One study in older pigs even suggested that repeated mixing might improve pigs' social skills and reduce aggression (88). We do not have indications that this was the case, though, as aggression did not decrease over the four different mixings. Third, the repeated mixings may not have been enough to result in allostatic overload in this study, as the stress from mixings was possibly too mild and short-lived. In contrast with the above mentioned studies describing long-term adverse effects of mixing in pigs (89), Merlot et al. (17) reported that endocrine and immune consequences of mixing in weanling piglets were temporary and absent on the long-term. If so, the repeatedly mixed pigs in our study may have coped with and recovered from mixing before the next regrouping occurred. Potentially, this successful coping with mixing prepared pigs for the challenges to come as it has been found that (predictable) mild stress, unlike chronic allostatic overload, might help animals to cope with future challenges (90, 91). It has been suggested that mild stress caused by adverse experiences in early life may increase survival and resilience in later life, as these experiences provide indications and forewarnings of the most likely future conditions (91, 92). For instance, a predictable chronic mild stress procedure (5 min of daily restraint stress for 28 days) in the early life of rats was found to enhance resilience against depression and anxiety caused by stress in later life (93). Finally, the mixed pigs may also been habituated to entering novel pens (which happened at mixing), making the novel pen in the Frustration test less stressful for them. This may have counteracted potential mixing effects on response in this test.

Repeatedly mixed pigs kept in barren pens showed a lower cortisol response to the LPS challenge than minimally mixed barren pigs, whereas such an effect was absent in enriched pigs. We do not have indications that barren pigs responded less vigorously to mixing; in contrast, they showed more aggressive acts than enriched pigs, and an increase of ear and tail biting over mixing events. Possibly, the repeated mixing may have had some positive aspects for the barren pigs, which had limited space and stimuli in their home pens to fulfill their behavioral needs. Prior to each mixing event, pigs were shortly kept in the corridor, which was more spacious than the pigs' pens. Also the relocation may have provided some new sensory stimuli to the barren pigs that were kept under generally stimulus-poor conditions.

### Effects on Natural Antibodies

Natural (auto)antibodies bind antigens without known exposure to these antigens. They are important as first line of immune defense and play a role in clearing apoptotic cells and maintaining B cell homeostasis (94–96). IgG antibodies initially decreased from week 3 to week 6, while the IgM antibodies increased over weeks. The IgG antibodies are in the first weeks of life likely derived from the mother, whereas the IgM antibodies largely represent the piglets' own synthesis, as sow colostrum and milk contain mainly IgG antibodies (97). KLH is a large glycoprotein with many epitopes, and higher KLH antibody titres have been associated with better disease resilience in chickens and pigs (4, 38, 39). A previous study reported higher titers of IgM and IgG binding KLH in pigs kept enriched with straw bedding from weaning onwards as compared with barren housed pigs (72), and demonstrated an increase in KLH antibody titres following regrouping, which was, for the IgG isotype, stronger in the enriched housed pigs. This is in line with our finding that repeatedly mixed enriched pigs showed higher KLH antibody titers than two of the other groups. It should be noted, though, that in other studies, in which enrichment was applied from birth, no or opposite results of enrichment were found (46, 98). Phosphorylcholine (PC) is recognized by natural autoantibodies after cell damage and inflammation (96) and a previous study found an increase in IgM binding PC-BSA shortly following regrouping (99), whereas IgG declined. This seems to be in line with the tendency we found for higher PC-BSA-IgM in repeatedly mixed piglets as compared with their minimally mixed counterparts, as well as the sharper decline in PC-BSA-IgG over time in repeatedly mixed pigs. Thus, the enrichment and mixing treatments applied in this study influenced natural (auto)antibodies, but the implications of these effects for pigs' health needs to be elucidated.

## Conclusion

In this study, enrichment did not enhance the speed of recovery from challenges in pigs, although there were indications of reduced stress. Enriched housed pigs did show a more favorable response to challenges and showed less ear and tail biting and less aggression in response to mixings. Repeated as opposed to single mixing did not seem to aggravate the negative effects of barren housing.

## Data Availability Statement

The raw data supporting the conclusions of this article will be made available by the authors, without undue reservation.

## Ethics Statement

The animal study was reviewed and approved by the Animal Care and Use Committee of Wageningen University & Research.

## Author Contributions

LL did the animal experiment and lab work, data analysis, and wrote the manuscript. LZ designed the experiment and did the animal experiment. MM did the animal experiment. SP designed the experiment and did part of the experimental work. JB designed the experiment and participated in data analysis. TR and EK were involved in designing the experiment. All authors involved in manuscript writing, read and approved the final manuscript.

## Funding

This study was part of the research project SmartResilience: toward a sustainable, future-oriented pig production system that supports and predicts resilience in pigs, with project number ALWGR.2017.007. The project is financed by the Netherlands Organization for Scientific Research (NWO), and Topigs Norsvin.

## Conflict of Interest

EK was employed by Topigs Norsvin. The remaining authors declare that the research was conducted in the absence of any commercial or financial relationships that could be construed as a potential conflict of interest.

## Publisher's Note

All claims expressed in this article are solely those of the authors and do not necessarily represent those of their affiliated organizations, or those of the publisher, the editors and the reviewers. Any product that may be evaluated in this article, or claim that may be made by its manufacturer, is not guaranteed or endorsed by the publisher.

## Footnotes

^1^Parois SP, Van Der Zande LE, Knol EF, Kemp B, Rodenburg TB, Bolhuis JE. A multi-suckling system combined with an enriched housing environment during the growing period promotes resilience to various challenges in pigs. *Sci Rep*. (under review).
